# Effects of Verapamil and Two Bisbenzylisoquinolines, Curine and Guattegaumerine Extracted from *Isolona hexaloba,* on the Inhibition of ABC Transporters from *Pseudomonas aeruginosa*

**DOI:** 10.3390/antibiotics11050700

**Published:** 2022-05-21

**Authors:** Christian Hulen, Pierre-Jean Racine, Marc Feuilloley, Abdelhakim Elomri, Nour-Eddine Lomri

**Affiliations:** 1 Bacterial Communication and Antimicrobial Strategies Research Unit, University of Rouen Normandy, 55 Rue Saint Germain, 2700 Evreux, France; hulen.marie@orange.fr (C.H.); pierre-jean.racine@univ-rouen.fr (P.-J.R.); marc.feuilloley@univ-rouen.fr (M.F.); 2 UNIROUEN, INSA Rouen, CNRS, COBRA (UMR 6014), Normandie University, 76000 Rouen, France; hakim.elomri@univ-rouen.fr; 3 Department of Biology, UFR Sciences and Techniques, University of Cergy-Pontoise, 2 Ave A. Chauvin, 95302 Cergy-Pontoise, France

**Keywords:** curine, guattegaumerine, verapamil, bacterial ABC transporters, antibiotics, MDR, antibiotic resistance, multidrug resistance

## Abstract

The biological effects of alkaloids, curine, guattegaumerine, and verapamil, on *Pseudomonas aeruginosa* were investigated. These molecules did not inhibit *P. aeruginosa* growth but increased the sensitivity of this bacterium to carbenicillin, novobiocin, and erythromycin. The results of another study indicate that curine and guattegaumerine were competitors of verapamil and acted as inhibitors of eukaryotic ABCB1 efflux pump. A BLAST-P carried out between a bacterial MDR transporter LmrA from *Lactococcus lactis*, a human MDR1/P-glycoprotein (ABCB1), and ABC proteins of *P.* *aeruginosa* highlighted five potential candidates that have this bacterium. A study on the sensitivity to carbenicillin in the presence of verapamil allowed us to identify the product of gene PA1113 as the ABC transporter involved in the influx of carbenicillin. Similarly, novobiocin transport performed in the presence of verapamil and a docking analysis highlighted protein MsbA (Lipid A flippase, gene PA4997) as a potential candidate in novobiocin efflux. MsbA has previously been identified as a multidrug transporter in *E. coli*, and as *P. aeruginosa* MsbA presented 76% identity with *E. coli* MsbA, it is possible that novobiocin efflux involves this ABC transporter, accounting for about 30% of the bacterium resistance to this antibiotic.

## 1. Introduction

*Pseudomonas aeruginosa* is an adaptable bacterial saprophyte found in soil, water, sewage, and plants. It is frequently defined as an opportunistic human pathogen causing many illnesses including tissue injury, burn wounds, and severe lung infection in cystic fibrosis patients [1,2]. It is also one of the common pathogens found in nosocomial pneumonia, urinary tract, and corneal infections [3]. A major problem in bacterial infection is that these organisms harbor natural and acquired resistance to many antibiotics. *P. aeruginosa* was identified by the World Health Organization as one of the three principal microorganisms regarding antibiotic resistance [4] and is one of the six ESKAPE pathogens with the highest risk of mortality, particularly in developing countries [5]. The multiple resistance of *P. aeruginosa* is mainly caused by low outer membrane permeability and the expression of efflux pumps. The major outer membrane porin OprF could be considered as the most important porin responsible for the large exclusion limit harbored by *P. aeruginosa* [6,7]. However, the loss of outer membrane permeability is not sufficient to explain the multi-resistance to antibiotics. 

The natural resistance of *P. aeruginosa* is also associated with the presence of effective efflux pumps expressed constitutively or induced under antibiotics pressure [8]. Six RND (Resistance Nodulation Division) systems have been identified in *P. aeruginosa*. The major system MexAB-OprM is frequently expressed in wild-type strains [9]. The other systems, MexCD-OprJ, MexEF-OprN, MexJK-OprM, and MexXY-OprM, are often silent in wild-type strains and are variably expressed in clinical isolates [10,11]. Recently the MexGHI-OpmD system, involved in the efflux of xenobiotics, heterocycle dye acriflavine, and norfloxacin, has been identified [12]. *P. aeruginosa* genome analysis also showed that this bacterium possesses three other multidrug efflux systems: the major facilitator superfamily (MFS), the small multidrug resistance family (SMR), and the multi-antimicrobial extrusion protein family (MATE) [13]. The strong resistance to antibiotics developed by *P. aeruginosa* requires the implementation of new strategies to fight against this bacterium [14].

Plants are an extraordinary reservoir of molecules that exhibit various biological activities on many different organisms. Curine and guattegaumerine are two alkaloids that we have isolated from the roots of *Isolona hexaloba*, Engl and Diels, one of the seven species growing in the humid forest of central Africa (Gabon). Triacetylguattegaumerine is a synthetic compound derived from guattegaumerine [15]. This family of molecules is known for its in vitro cytotoxicity as well as for its antiplasmodial and amoebicidal activities [16,17]. Curine is also characterized as a molecule with calcium pump inhibitory activity and as a vasodilatory alkaloid [18]. It has been shown that curine has a potent cytotoxic effect on leukemic cell line HL60 by cell cycle arrest and apoptosis induction [19]. Curine also exhibits anti-allergic effects in mouse models of asthma, skin, and systemic allergies [20]. More recently, Ribeiro-Filho et al. [21] demonstrated that curine inhibits cytokine secretion, including tumor necrosis factor (TNFα), interleukin (IL) -1β, IL-6 and nitric oxide (NO) production in lipopolysaccharide (LPS)-activated macrophages by reducing Ca^2+^ influx with the same efficiency as verapamil, a known inhibitor of drug efflux pumps [22]. The pharmacological activity of guattegaumerine has not yet been well established, although 30 years ago, it was suggested to have antimitotic activity [23]. Only recently was guattegaumerine shown to have a protective activity against oxidative stress injury in primary cortical neurons [24]. 

The results presented in our submitted article [15] demonstrate the inhibitory effect of these bisbenzylisoquinolines on Mdr1b/P-gp (ABCB1), the major drug efflux protein belonging to the ABC transporter family in animal cells. We also show that these alkaloids are competitors of verapamil, a strong inhibitor of this protein, for binding to Mdr1. Herein, we show the effects of curine, guattegaumerine, and verapamil on the growth of two strains of *P. aeruginosa* and on the sensitivity of this bacterium to different antibiotics potentially expelled through the Mex-family dependent systems.

## 2. Results

### 2.1. Evaluation of the Response of Pseudomonas Aeruginosa to Antibiotic Exposure in the Presence of Curine, Guattegaumerine, and Verapamil

**Figure 1:**
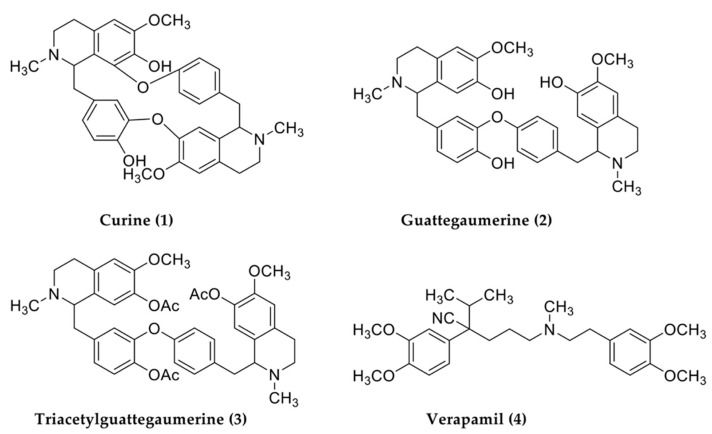
Structures of alkaloids used in this study. Curine and guattegaumerine were purified from the crushed roots of Isolona hexaloba. The structure of guattegaumerine was resolved through acetylation of the compound to give triacetylguattegaumerine. Verapamil was of commercial origin (Sigma).

In addition to our investigation of the medical potential of West African plants [25,26], we purified two bisbenzylisoquinolines, curine and guattegaumerine, from the roots of *Isolona hexaloba*, and realized the hemi-synthesis of triacetylguattegaumerine to determine the chemical structure of guattegaumerine (Figure 1). This family of molecules is known for its in vitro cytotoxicity as well as for its antiplasmodial and amoebicidal activities. Some of these molecules are known for their impact on bacterial growth [16,17]

**Figure 2:**
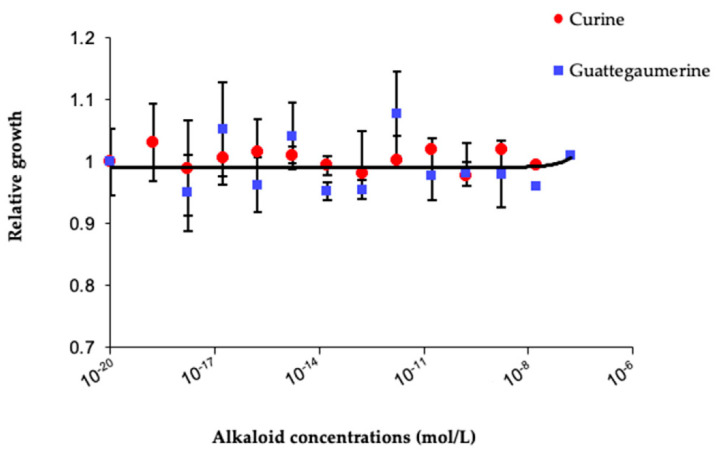
Effects of curine and guattegaumerine on *P. aeruginosa* growth. A total of 100 µL LB medium containing 1 × 10^5^ bacteria of strain IP104116 and increasing concentrations of curine or guattegaumerine were added in each well of 96-well plates. After 24 h of incubation at 37 °C in a wet chamber, growth was estimated by measuring the absorbance at 595 nm and was compared to the control without curine or guattegaumerine and plotted against concentration in alkaloids in the corresponding well. Experiments were repeated at least four times for each strain of *P. aeruginosa* used in this study.

Therefore, the effects of curine and guattegaumerine on *P. aeruginosa* bacterial growth were investigated. To explore these effects, two strains were used: one non-mucoid from a collection of cultures (strain IP104116) and the other typical mucoid isolated from the lung of a patient with cystic fibrosis (strain NK125502). As shown in Figure 2, curine and guattegaumerine did not inhibit bacteria growth in a range of concentrations from 1 × 10^−19^ M to 1 × 10^−6^ M.

These molecules are known for their inhibitory activity on calcium pumps as well as on certain MDR proteins of the ABC transporter family in eukaryotes [15]. Therefore, we examined the effects of curine, guattegaumerine, and triacetylguattegaumerine on the sensitivity of *P. aeruginosa* (NK125502 and IP104116) to three antibiotics (carbenicillin, novobiocin, and erythromycin), which belong to three different families of antibiotics. These three antibiotics act on different targets in the bacterium:

- Carbenicillin on periplasmic penicillin-binding proteins to block peptidoglycan synthesis;
- Novobiocin on gyrase B-subunit by binding to the ATP-binding site to block DNA synthesis and transcription;
- Erythromycin on the 50S ribosomal sub-unit to block protein synthesis.

**Figure 3:**
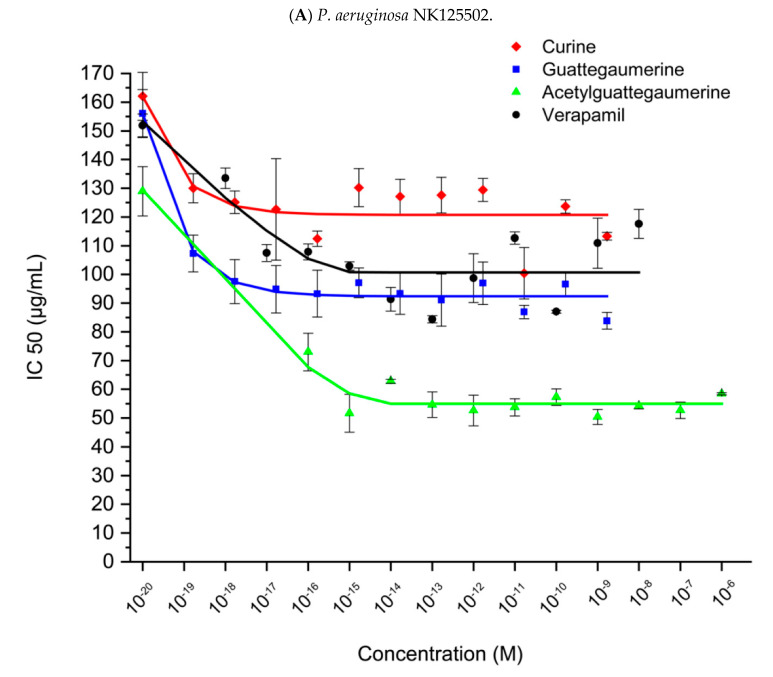

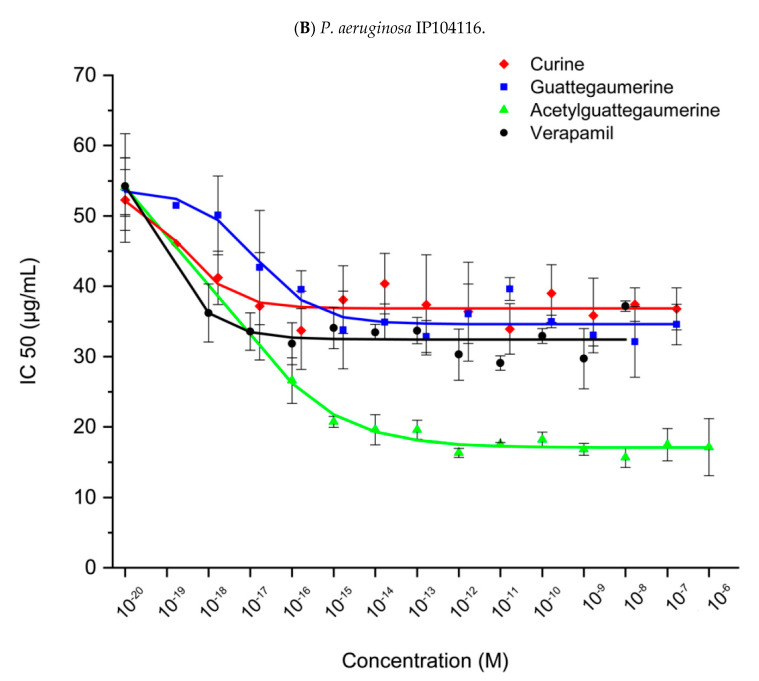
Effects of bisbenzylisoquinolines and verapamil on the sensitivity of *P. aeruginosa* NK125502 (clinical strain) (**A**) and IP 104116 (Pasteur Institute) (**B**) to carbenicillin.

The decrease in IC50 values observed in Figure 3 was the result of the action of the alkaloids on bacteria, rendering them more sensitive to carbenicillin and inducing a considerable decrease in viable bacteria in the inoculums.

Bacteria were inoculated in 96-well plates with increasing concentrations of alkaloids (lines) and carbenicillin (columns) in 100 µL LB medium. After 24 h of incubation at 37 °C, bacterial growth was estimated in each well and reported as growth without antibiotics for each alkaloid concentration. Relative growth was plotted against concentration in carbenicillin and IC50 was calculated for each concentration in alkaloids from the equation of the curve. Then, the obtained values were plotted against concentration in alkaloids (curine, guattegaumerine, tri-acetylguattegaumerine, and verapamil). The results are the mean values of at least four independent experiments. 

Figure 3A presents the IC50 variation in the clinical strain NK125502 after exposure to carbenicillin with increasing concentrations of curine, guattegaumerine, triacetylguattegaumerine, and verapamil. An increase in the sensitivity to the antibiotic was observed. The presence of the exopolysaccharide alginate did not prevent the bisbenzylisoquinolines from reaching their targets. 

Figure 3B shows an example of IC50 variation in the IP104116 strain after exposure to carbenicillin in the presence of the same alkaloids. It can be noted that the resistance of strain IP104116 to carbenicillin is reduced in the presence of alkaloids in the culture medium to reach a plateau for a concentration of 1 × 10^−15^ M. Beyond this concentration, the sensitivity to the antibiotic did not vary significantly, thus demonstrating the presence of a saturable system sensitive to these alkaloids.

The decrease in IC50 values observed in Figure 3 was the result of the action of the alkaloids on the bacteria, which made them more sensitive to carbenicillin and induced a considerable decrease in the number of viable bacteria in the inoculum.

**Figure 4:**
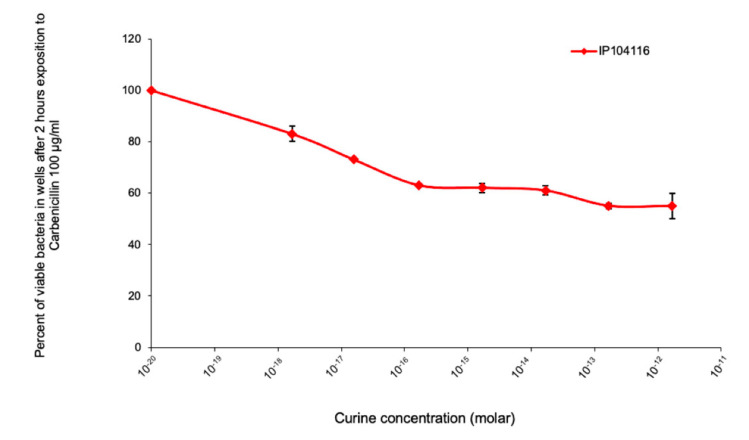
Effects of curine on the sensitivity of *P. aeruginosa* (IP104116) to carbenicillin.

As an example, Figure 4 presents the variation in the number of viable bacteria after 2 h of exposure to carbenicillin in the presence of curine. Increasing concentrations of curine in the culture medium containing 100 µg/mL of carbenicillin induced a decrease in the number of viable bacteria (with a maximum loss of 40% for a curine concentration of 1 × 10^−15^ M) in the same order of magnitude as the increase in the susceptibility of the bacterial strain to this antibiotic. 

A total of 50 µL of LB in the presence or absence of 200 µg/mL of carbenicillin and increasing concentrations of curine was added to 50 µL of LB containing about 2 × 10^5^ bacteria in a 96-well plate. After two hours of incubation at 37 °C, viable bacteria were numbered by plating the adequate dilution on LB agar plates. The percentage of viable bacteria was calculated and plotted as a function of the curine concentration. The results presented in this figure are the mean values of three independent experiments.

Curves presenting the variation in IC50 in the presence of the alkaloids obtained for the two strains of *P. aeruginosa* for each antibiotic had the same shape. For a simplified analysis, a calculation of the average value of the IC50 at the plateau was performed, and the results are presented in Table 1 and Table 2.

The average value of the IC50 at the plateau (calculated from each curve, as presented in Figure 3A) for each antibiotic was reported as a function of each alkaloid and compared to the value of IC50 in the absence of alkaloids. Experiments were repeated at least four times for each alkaloid. *p*-values were obtained using Fisher’s exact test.

It can be noted that the strain IP104116 is moderately resistant to carbenicillin with an IC50 of 58 mg/L, and is resistant to novobiocin (IC50 = 289 mg/L) as already indicated in the literature [8,21], but it is rather sensitive to erythromycin (40 mg/L). 

Table 1 also shows a reduction in the IC50 values for each antibiotic in the presence of each alkaloid. This reveals a greater sensitivity of the IP104116 strain to the antibiotics tested. It may be noted that the treatment with the alkaloids (first column) increases susceptibility to carbenicillin by 36% with curine, 28% with guattegaumerine, and 41% with triacetylguattegaumerine. The same effects of the alkaloids were observed in terms of the sensitivity to novobiocin (curine 40%, guattegaumerine 37%, and triacetylguattegaumerine 28%) and sensitivity to erythromycin (curine 25%, guattegaumerine 45%, and triacetylguattegaumerine 25%).

By analogy with eukaryotic cells, we also investigated the effects of verapamil, an inhibitor of ABC transporters, on the sensitivity of *P. aeruginosa* to antibiotics. With the strain IP104116, we noted (Table 1) an increase in the sensitivity to antibiotics in the presence of verapamil with a 40% gain in sensitivity to carbenicillin, 34% to novobiocin, and 24% to erythromycin. The three bisbenzylisoquinolines and verapamil appeared to block the ABC transporters involved in the transport of these antibiotics.

Treatment of the NK125502 strain by alkaloids had similar effects, with a global increase in sensitivity, except for erythromycin (Table 2). Sensitivity to carbenicillin was increased by 40% in the presence of curine, 42% with guattegaumerine, 50% with triacetylguattegaumerine, and 40% with verapamil. Similarly, the sensitivity to novobiocin was increased by 18% with curine, 27% with guattegaumerine, 16% with triacetylguattegaumerine, and 23% with verapamil. These decreased effects of alkaloids on the NK125502 strain’s sensitivity to novobiocin are often associated with the expression of several Mex systems in clinical strains [8,9]. In addition, the alkaloids had no effect on the sensitivity of this strain to erythromycin because this antibiotic can be transported by different Mex pumps, AB, CD, and XY [27]. Since clinical strains might express several of these systems, the action of alkaloids on ABC transporters should not impair RND systems.

### 2.2. Identification of Carbenicillin and Novobiocin Transport Proteins in Pseudomonas Aeruginosa PAO1

Firstly, we implemented an in silico approach based on amino acid sequence homologies between proteins identified as targets of verapamil and of our bisbenzylisoquinolines: ABCB1 (P-Gp Homo sapiens) [28], Abcb1b (Mus musculus) and LmrA from *Lactococcus lactis*, which is capable of replacing the human multidrug-resistance P-glycoprotein gene ABCB1 in human KO cells [29], BmrA from *Bacillus subtilis* [30] and ABC transporters of *P. aeruginosa*. After recovering the amino acid sequences of ABCB1, Abcb1a, LmrA, and BmrA, we performed BLAST-P against *P. aeruginosa* PAO1 proteins. From their higher scores and multiple appearances in BLAST results, five ABCs out of the hundred found in PAO1 were identified as potential targets for our alkaloids. In the order of genes, on the chromosome, we have PA0860, PA1113, PA3228, PA4143 (possible transport of toxins and bacteriocins), and PA4997 (lipid A transporter MsbA, a flippase). From the Washington UC collection, we obtained mutants in each of these genes except for PA4997 and the isogenic wild-type strain PAO1. These mutants and PAO1 were tested for their sensitivity to carbenicillin and novobiocin in the presence of verapamil as a representative inhibitor of our alkaloids (Table 3).

First of all, it should be noted that the wild-type reference strain exhibits a lower sensitivity to carbenicillin by more than half of that of its mutants. The formation of mutants by the insertion of a transposon carrying a kanamycin resistance gene potentiates the bacterium by activating the efflux systems and, in particular, the Mex systems, probably by acting on the expression levels of MexS and MexT [31]. This explanation also applies to the differences observed with novobiocin.

Table 3 shows the maximum variations in IC50 for carbenicillin and novobiocin for the different mutants and *P. aeruginosa* PAO1 in the presence of verapamil (1 × 10^−15^ M and more). With carbenicillin (Table 3, left), only mutant PW3010 showed no change in antibiotic sensitivity in the presence of verapamil. It seems that this mutant lost the ABC transporter, the PA1113 gene product, responsible for carbenicillin transport through the cytoplasmic membrane, inducing part of the resistance of the bacterium to carbenicillin. Further analyses have shown that this ABC transporter mediates carbenicillin uptake [32].

The exposure of *P. aeruginosa* PAO1 and the different mutants to novobiocin in the presence of verapamil showed a constant increase in the susceptibility of bacteria to this antibiotic (Table 3, right). None of the mutants tested, despite selection among ABC transporters likely to be the target of verapamil, lost their increased sensitivity to novobiocin in the presence of verapamil. The only candidate that was not tested was PA4997, since the loss of the lipid A output from the cytoplasm should be lethal for the bacterium [33,34].

**Figure 5:**
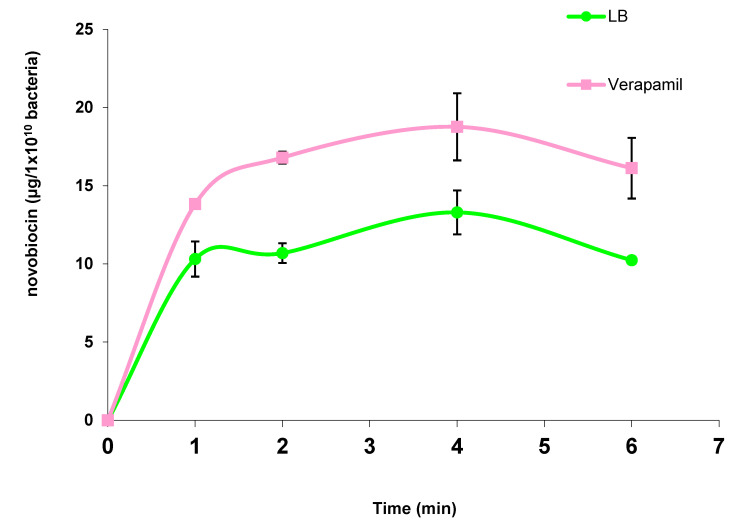
Kinetics of accumulation of novobiocin in the cytoplasm of *P. aeruginosa* PAO1 in the absence or presence of verapamil.

Therefore, we verified that verapamil does have an effect on one of the PAO1 ABC transporters involved in the transport of novobiocin. Figure 5 shows the kinetics of the accumulation of novobiocin in the cytoplasm of *P. aeruginosa* PAO1 in the presence and absence of verapamil.

*P. aeruginosa* PAO1 was suspended in LB medium or LB + 1 × 10^−7^ M verapamil at a concentration of 1 × 10^10^ bacteria per ml and incubated for 20 min at 37 °C before starting kinetics, performed as described in the Materials and Methods. The bacteria were then lysed by sonication and the cytoplasmic contents recovered after centrifugation. The amount of novobiocin in cytoplasmic extracts was determined from a standard range established with the sensitive bacterium *Bacillus subtilis* 168 and plotted against time. The results presented in Figure 5 are the mean values of at least three independent experiments.

We observed that bacteria treated with verapamil at a concentration of 1 × 10^−7^ M had about 35% more novobiocin in their cytoplasm than untreated bacteria, the same order of magnitude as the increase in the sensitivity. Since the target of novobiocin is the intracytoplasmic protein gyrase, it can be assumed that verapamil acts on an ABC transporter involved in the efflux of this antibiotic. In addition, the ABC efflux transporter inhibited by verapamil was not present in the mutants tested in these studies. Therefore, one of the possibilities is that the novobiocin is exported by the MsbA gene product.

**Figure 6:**
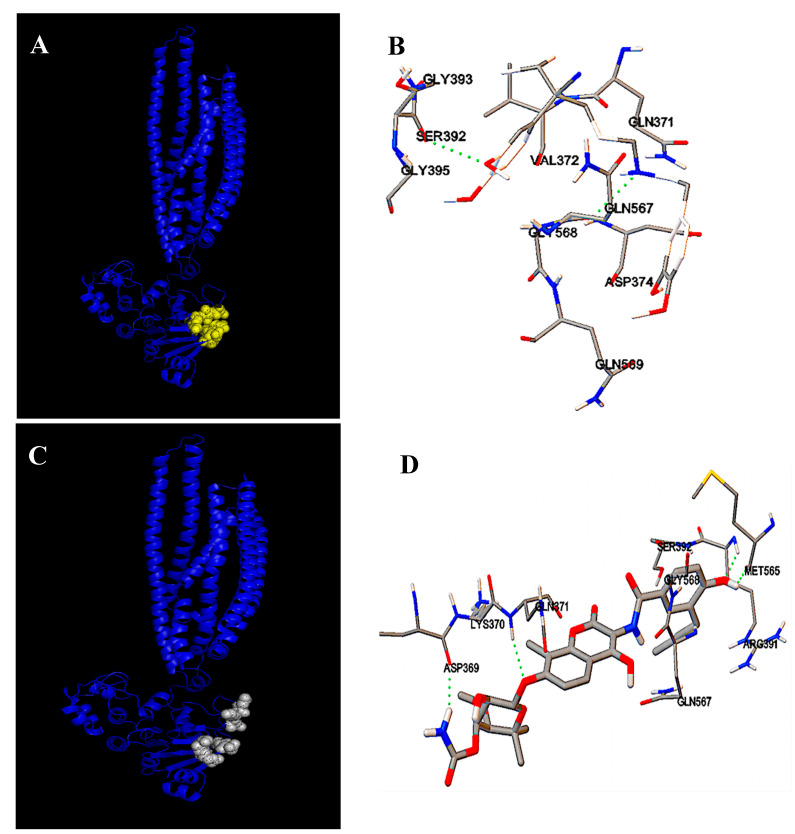
Molecular modelization and docking analysis of protein MsbA interactions with verapamil and novobiocine. (**A**) shows the modeled interactions of the MsbA ABC transporter with verapamil; (**B**) shows in green dotted lines the amino acids involved in the interaction of MsbA with verapamil; (**C**) shows the modeled interactions of the MsbA ABC transporter with novobiocin; and (**D**) shows in green dotted lines the amino acids involved in the interaction of MsbA with novobiocine.

The molecular modelization of protein MsbA followed by a docking analysis revealed that novobiocin and verapamil should interact at a common site on the protein (Figure 6).

The interaction site involves the amino acids Asp369, Lys370, Gln371, and Val 372 located upstream of the Walker A motif, and the amino acids Arg391, Ser392, and Gly393 located in the Walker A motif. The amino acids Met565, Gln567, Gly568, and Gln569 are also involved in the interaction of novobiocin and verapamil with MsbA and are located downstream of the Walker B motif. Additionally, the amino acids Gly 434, Leu 435, Arg507, and Lys511 in the ATP binding site are involved. The modeling software gives free energy values for the fixation of the two molecules, ΔG_0_ = −5.85 kcal/mole and −6.9 kcal/mole for verapamil and novobiocin, respectively, and −7.75 kcal/mole for ATP. According to this analysis, novobiocin should be extruded from the cytoplasm of PAO1 by MsbA and verapamil should act as a competitive inhibitor.

This hypothesis is consistent with the observations made with LmrA *L. Lactis* and MsbA from *E. coli* [35], that they are similar proteins with high percentages in terms of identity (Table 4).

## 3. Discussion

The genus *Isolona* is rich in natural molecules, including several families of alkaloids. Several species in the genus *Isolona* have been used for the extraction of therapeutic molecules [36], but for the first time, we purified two bisbenzylisoquinolines, curine (1) and guattegaumerine (2), from the roots of *Isolona hexaloba* (Figure 1). Physical and spectral analyses were used to determine the structure of curine. For guattegaumerine, the resolution could only be achieved after hemi-synthesis of the tri-acetylated derivative form (3) (Figure 1). These molecules have been previously tested for their therapeutic potential, essentially antiparasitic, antiplasmodial, and antimitotic activities [16,17]. Similarly, curine has been previously tested as a vasodilator compound, and as an inhibitor of calcium pumps [18], and was found to be cytotoxic on leukemic HL60 cell lines [19].

In this study, we show that curine and guattegaumerine are not toxic to *P. aeruginosa* in a wide range of concentrations (10^−19^ to 10^−6^ M). Treatment of the non-mucoid strain IP104116 by these alkaloids and verapamil caused increased sensitivity of this strain to the three antibiotics, carbenicillin, novobiocin, and erythromycin, tested in this study. The mucoid clinical strain NK125502 presented the same response to treatments with alkaloids, except for erythromycin, but this difference can be easily explained by the great diversity of the efflux systems existing for this antibiotic in clinical strains [10,27]. Like many bacteria responsible for nosocomial infections, *P. aeruginosa* is naturally resistant to many antibiotics, particularly through the presence of nine efflux systems assigned to the family RND (resistance nodulation cell division), including the six Mex-systems which confer significant resistance to antibiotics [11,12,37].

Alkaloids used in this study were identified as inhibitors of ABC transporters in animal cells. As the treatment of *P. aeruginosa* with these alkaloids makes the bacteria more susceptible to antibiotics belonging to three different families, bacterial ABC transporters seemed to be involved in setting the resistance of *P. aeruginosa* to antibiotics. Using the amino acid sequences of eukaryotic proteins identified as targets of our alkaloids, ABCB1 and two ABC transporters from Gram+ bacteria, LmrA [29,38] and BmrA [39], we performed a BLAST-P against the total proteins of *P. aeruginosa* PAO1. We then identified five potential candidates among the hundreds of ABC transporters that have this bacterium.

By focusing our study on sensitivity to carbenicillin in the presence of verapamil, we found that the mutant of the gene PA1113 no longer presented any variation in sensitivity to the antibiotic, unlike the isogenic wild strain. The PA1113 gene product has been identified as the target for verapamil and our alkaloids. This ABC transporter is implicated in the uptake of carbenicillin in the cytoplasm and it is responsible for about 40% of PAO1 resistance to this antibiotic [32].

The results obtained with novobiocin in the presence of verapamil are more complex to interpret. First, treatment of PAO1 with verapamil showed an intracytoplasmic accumulation of novobiocin. Therefore, it seems that verapamil would act on the efflux of the antibiotic by blocking an ABC transporter. However, the treatment by verapamil of mutants in four candidate genes increased the sensitivity to novobiocin, providing evidence that the target protein was still functional in each of these mutants. For the fifth candidate gene, PA4997, no mutant exists in the PAO1 Washington library of mutants. Because the product of this gene has been identified as a lipid A carrier, it is understandable that if lipid A cannot escape the cytoplasm of the bacteria and reach the outer membrane, there will be no more LPS in this membrane. Therefore, the membrane cannot be organized and the bacteria will die [35,40]. However, the PA4997 gene product was identified three times with good scores when BLAST-P was performed with the amino acid sequences of the eukaryotic target proteins ABCB1 (human) and abcb1 (rat) and the LmrA protein. One can hypothesize that the novobiocin efflux from the cytoplasm of PAO1 could be carried out by the ABC transporter MsbA, but in the absence of an available mutant, we will need another strategy to demonstrate such a hypothesis. A docking approach following the molecular modelization of MsbA showed that novobiocin and verapamil should bind to the protein at the same sites in the NBD domain. One site includes the Walker A motif (P-loop), and the other one, amino acid sequences downstream from the Walker B motif. Since verapamil and novobiocin seem to interact with MsbA at the same site, we could speculate on the competitive characteristics of verapamil with novobiocin.

Additionally, *Escherichia coli* MsbA presents a high homology with the multidrug transport ATP-binding cassette protein LmrA (75%). It has been shown that in *E. coli*, MsbA is an efflux transporter for erythromycin, monoglyceride lipid monomyristin, ethidium bromide, and Hoechst 33342 [40]. Ethidium and Hoechst 33342 transport revealed apparent single-site kinetics with competitive inhibition by vinblastin, which is a known ABCB1 inhibitor [40]. It has also been shown that MsbA can bind the anticancer molecule daunorubicin at the cytoplasmic end of helices 3 and 6 into an aqueous chamber formed at the interface between the two transmembrane domains [41]. The results of cysteine cross-linking, fluorescence energy transfer and cysteine accessibility studies on positions near the nucleotide-binding domains and in the membrane domains with *E. coli* MsbA suggest that substrate binding stimulates the maximum rate of ATP hydrolysis by facilitating the dimerization of nucleotide-binding domains [42]. *P. aeruginosa* MsbA presents 76% identity with *E. coli* MsbA (Table 4). This suggests that MsbA from *P. aeruginosa* should be an MDR transporter involved in the efflux of novobiocin and in the flip of lipid A as its principal function. In addition, the analysis of a highly novobiocin-resistant mutant in *E. coli* brings new information on the bacterial resistance to this antibiotic. J.M. May et al. have shown that novobiocin directly binds the ATPase LptB subunit of the transmembrane LPS transporter. This interaction increases the activity of the LPS transporter and decreases the outer membrane permeability [43]. All of these membrane proteins, MsbA, the LPS transporter, and the RND proteins belonging to the Mex system, each at their own level, help establish the bacteria’s resistance to novobiocin. Regarding MsbA, it appears that its participation in the overall resistance of *P. aeruginosa* to the antibiotic is around 35%.

## 4. Materials and Methods

### 4.1. Plant Materials

Roots of *Isolona hexaloba* Engl. & Diels (Annonaceae) were harvested in the locality of Sibang, near Libreville, east of Gabon, in February 2003. A voucher sample (#2082) was deposited in the National Herbarium of Gabon in Libreville. Samples were dried and powdered in the shade, at room temperature. 

### 4.2. Chemicals

All chemicals used in this study to extract and purify alkaloids were research-grade. Verapamil, carbenicillin, novobiocin, and erythromycin were obtained from Sigma-Aldrich Chimie S.a.r.l (St. Quentin Fallavier, France).

### 4.3. Alkaloidic Extraction and Isolation of the Bisbenzylisoquinolines

To isolate the alkaloids, 225 g of root powder was submitted to a procedure aimed at isolating alkaloids by using solvent extraction, column, and thin-layer chromatography. The complete procedure was described in our work submitted for publication [15].

### 4.4. Triacetylguattegaumerine (3) Preparation

To a solution of guattegaumerine was added an excess of acetic anhydride. The reaction mixture was stirred at room temperature for 24 h and then diluted with cold water, extracted with CH_2_Cl_2_, the organic layer dried over Na_2_SO_4_, filtered, and the solvent evaporated under vacuum to yield the title compound as a brown solid [15].

### 4.5. Molecular Structure Determination

The structure of isolated compounds was determined by physical and spectral data analyses [24]. The structure of guattegaumerine was actually confirmed owing to the synthesis and spectral analysis of triacetylguattegaumerine (3) [15]. These structures (Figure 1) were in agreement with those published in the literature [44,45]. 

### 4.6. Bacterial Strains

*Pseudomonas aeruginosa* strain 175CIP104116 (serotype O) was obtained from the Institut Pasteur collection. 

*P. aeruginosa* strain PAO1 and the mutants PW2567, PW3010, PW6408, and PW8020 were from the Washington UC collection.

*P. aeruginosa* NK 125502 was from our laboratory collection. This mucoid strain was isolated a long time ago from a cystic fibrosis patient’s lung at the Hospital Necker, Paris, France, and was a gift from Pr. P. Berche.

*Bacillus subtilis* 168 was from our collection.

Strains were stored at −80 °C in LB containing 20% glycerol. Bacteria are routinely grown in Luria broth (LB) medium and on LB agar plates. 

### 4.7. Sensitivity to Antibiotics

Solutions of curine, guattegaumerine, triacetylguattegaumerine, and verapamil were prepared in LB at concentrations twofold of the final concentration used, between 1 × 10^−20^ and 1 × 10^−9^ molar. For varying concentrations of antibiotics (carbenicillin, novobiocin, and erythromycin) 96-well plates containing 100 µL per well were used to measure bacterial growth in the absence and presence of the four drugs (50 µL of LB with 2 × 10^5^ bacteria plus 50 µL of LB containing alkaloids and antibiotics). In each plate, the first row did not contain antibiotics but did contain increasing concentrations of alkaloids to measure maximal growth and drugs’ toxicity. In addition to alkaloids, the wells of the following rows contained increasing concentrations of antibiotics for each column. At t_0_ the absorbance at 595 nm in each well was measured in a plate reader µQuant (Bio-Tek instruments, Inc.) and the plates were incubated for 24 h at 37 °C in a wet chamber. After incubation, bacterial growth was estimated from the absorbance at 595 nm. For each concentration of drug and antibiotic, the relative growth was calculated as the ratio of the absorbance value in the wells to that of the control, and the results were plotted against the concentration of antibiotics. The minimal concentration of antibiotics that inhibits 50% of growth (IC50) was calculated from the equation of the curve for each concentration in drugs and plotted against the related drug concentrations. 

The effect of carbenicillin on viable bacteria in the inoculums was measured in the presence of increasing concentrations of curine. A total of 50 µL of LB in the presence or absence of 100 µg/mL carbenicillin and concentrations of curine in a range of 2 × 10^−18^ to 2 × 10^−12^ molar were added to 50 µL of LB containing 2 × 10^5^ bacteria in a 96-well plate and incubated for 2 h at 37 °C in a wet chamber. Viable bacteria were numbered by plating the adequate dilution on LB agar plates. The percentage of viable bacteria after treatment with carbenicillin in the presence of curine was calculated and plotted as a function of the curine concentration. 

### 4.8. Kinetics of Novobiocin Accumulation

*P. aeruginosa* PAO1 was grown to confluence on LB agar plates for 24 h at 37 °C. Bacteria were recovered by scraping with a sterile loop and suspended at a concentration of 1 × 10^10^ bacteria per ml, in LB medium or LB medium containing 1 × 10^−7^ molar verapamil. After 20 min of incubation at 37 °C to allow the interaction of verapamil with its targets, kinetics were performed for 1, 2, 4, and 6 min in Eppendorf tubes containing 250 µg of novobiocin per ml. At t + 6 min, the tubes were centrifuged for 3 min. at 10,000× *g* and at 4 °C to stop the transport of the antibiotic. The pellets were resuspended in 500 µL of sterile water for each tube and bacteria were broken by sonication in a Branson sonifier 6 times for 30 s on ice. Lysates were centrifuged for 20 min at 15,000× *g* at 4 °C and the cytoplasmic contents were recovered and filtered through 0.45 µm filters.

The presence of novobiocin in the cytoplasmic extracts was detected by the inhibition of the growth of *Bacillus subtilis* 168 as a control bacterium that is highly sensitive to novobiocin. Approximately 1 × 10^6^ *B. subtilis* 168 were plated on LB agar plates and incubated for 1 h at 37 °C. Wells of 5 mm in diameter were cut in the agar. Totals of 50 µL, 25 µL, 10 µL, and 5 µL of cytoplasmic extracts of each kinetic were added into the wells and the volume completed to 50 µL with sterile water. For each experiment, a plate containing 7 wells was used to determine the sensitivity of *B. subtilis* 168 to novobiocin and the construction of a standard curve between 100 ng and 1.5 µg per well. After overnight incubation at 37 °C, the diameter of growth inhibition areas around the wells was measured. With the control plate, a sensitivity calibration range to novobiocin is built from the width of the halos of inhibition (in mm) as a function of the amount of novobiocin per well (in µg) (Figure 2). For each experiment, the size of each halo is plotted on the reference range and the amount of novobiocin in each well is determined. For each point of the kinetic, values are expressed in µg novobiocin to 1 × 10^10^ bacteria.

### 4.9. Molecular Modelization

The FASTA amino acid sequences of LmrA *L. lactis* (access code P97046), MsbA *E.Coli* (access code P60752) and MsbA *P.aerugenosa* (access code Q9HUG8) were obtained from UniProtKB. The 3D structure of MsbA protein was calculated by RaptorX [46] and visualized using the open-source software Python Molecular Viewer V1.5.6 from Scripps Research Institute, La Jolla, CA, USA [47]. Potential interactions between ligands and the target protein were assayed using AutoDock 4 [48]. 

## 5. Conclusions

We have shown in this study that the use of ABC transporter inhibitors may increase the efficacy of antibiotics but not sufficiently to totally eliminate the bacteria. The research and development of new efflux pump inhibitors, obtained either by synthesis or extraction from nature, is a promising approach for the implementation of a combination therapy involving both pump inhibitors and antibiotics. In conclusion, we believe that it is virtually impossible to eradicate the opportunistic pathogenic bacteria responsible for chronic infections or sepsis when they are resistant or multi-resistant to antibiotics. However, by using a therapy directed against several bacterial targets, it should be possible to significantly reduce the microbial load so that the commensal flora and the immune system control the spread of infectious exogenous bacteria, as already evoked in the literature [49,50].

## Figures and Tables

**Table 1:** IC50 of *P. aeruginosa* IP104116 for carbenicillin, novobiocin, and erythromycin in the presence or absence of alkaloids.

	IC 50 (µg/mL)		
Strain IP 104116	Carbenicillin	Novobiocin	Erythromycin
	Mean ± SD, (*p*-Value)		
None Curine Guattegaumerine Triacetylguattegaumerine Verapamil	58 ± 3.5 37 ± 1.5, (*p* = 0.038) 42 ± 1, (*p* = 0.01) 34 ± 1, (*p* = 0.02) 35 ± 3.5, (*p* = 0.052)	289 ± 3.5 172.52 ± 4.94, (*p* = 0.14) 180.74 ± 7.28, (*p* = 0.16) 208.54 ± 2.43, (*p* = 0.09) 191.94 ± 2.26, (*p* = 0.19)	40 ± 4 29.86 ± 1.74, (*p* = 0.18) 22.39 ± 1.47, (*p* = 0.22) 29.85 ± 2.48, (*p* = 0.19) 30.60 ± 0.49, (*p* = 0.17)

**Table 2:** IC50 of *P. aeruginosa* NK125502 for carbenicillin, novobiocin, and erythromycin in the presence or absence of alkaloids.

	IC 50 (µg/mL)		
Strain NK 125502	Carbenicillin	Novobiocin	Erythromycin
	Mean ± SD, *p*-Value		
None Curine Guattegaumerine Triacetylguattegaumerine Verapamil	154.61 ± 13.5 105.27 ± 9.8, (*p* = 0.004) 95.79 ± 5.36, (*p* = 0.122) 82.06 ± 6.8, (*p* = 0.075) 101.05 ± 5.48, (*p* = 0.063)	24.77 ± 1.54 19.85 ± 0.93, (*p* = 0.13) 17.76 ± 2.18, (*p* = 0.12) 20.70 ± 0.91, (*p* = 0.16) 19.11 ± 0.90, (*p* = 0.11)	38.95 ± 3.43 38.16 ± 1.23, (*p* = 0.01) 38.80 ± 0.55, (*p* = 0.03) 36.82 ± 1.77, (*p* = 0.05) 39.81 ± 1.81, (*p* = 0.03)

**Table 3:** Effects of verapamil on the sensitivity of *P. aeruginosa* PAO1 and its mutants to carbenicillin and novobiocin.

		Carbenicillin IC 50 (µg/mL)		Novobiocin IC 50 (µg/mL)	
Strain	Gene	None	Verapamil	None	Verapamil
		Mean ± SD	Mean ± SD, *p*-Value	Mean ± SD	Mean ± SD, *p*-Value
PAO1 Washigton		29 ± 3.5	21 ± 2, (*p* = 0.05)	193 ± 3	166 ± 2, (*p* = 0.002)
PW2567	PA0860	nd	nd	267 ± 12	231 ± 9, (*p* = 0.015)
PW8020	PA4143	42 ± 2	23 ± 1, (*p* = 0.08)	180 ± 11.5	158.5 ± 6.5, (*p*= 0.002)
PW6408	PA3228	55 ± 4	39 ± 4, (*p* = 0.09)	249 ± 8	210 ± 5.5, (*p* = 0.09)
PW3010	PA1113	54 ± 2	54 ± 1.5, (*p* = 0.01)	146.5 ± 3.5	127 ± 3.5, (*p* = 0.10)

The average value of the IC50 at the plateau (calculated from each curve, as presented in Figure 4) for carbenicillin or novobiocin in the presence of verapamil was reported (right columns) and compared to the value of IC50 in the absence of verapamil (left columns). The results presented in this table are the mean values of at least four independent experiments. *p*-values (*p*) were obtained using Fisher’s exact test.

**Table 4:** Amino acid sequence alignment of LmrA *L.* *lactis* (P97046), MsbA *E.Coli* (P60752) and MsbA *P. aeruginosa* (Q9HUG8) using the freeware Serial Cloner software (RRID:SCR_014513).

Percentage of Identity			
	LmrA (*Lactococcus lactis*)	MsbA (*E. Coli*)	MsbA (*P. aeruginosa*)
LmrA (*Lactococcus lactis*)	100	75.18	77.33
MsbA (*E.Coli*)	75.18	100	76
MsbA (*P. aeruginosa*)	77.33	76	100
